# An integrative characterization of proline *cis* and *trans* conformers in a disordered peptide

**DOI:** 10.1016/j.bpj.2024.09.028

**Published:** 2024-09-27

**Authors:** Alice J. Pettitt, Vaibhav Kumar Shukla, Angelo Miguel Figueiredo, Lydia S. Newton, Stephen McCarthy, Alethea B. Tabor, Gabriella T. Heller, Christian D. Lorenz, D. Flemming Hansen

**Affiliations:** 1 Department of Structural and Molecular Biology, Division of Biosciences, London, United Kingdom; 2 Department of Chemistry, Faculty of Mathematical and Physical Sciences, London, United Kingdom; 3 Department of Engineering, Faculty of Natural, Mathematical and Engineering Sciences, King’s College London, London, United Kingdom; 4 The Francis Crick Institute, London, United Kingdom

## Abstract

Intrinsically disordered proteins (IDPs) often contain proline residues that undergo *cis/trans* isomerization. While molecular dynamics (MD) simulations have the potential to fully characterize the proline *cis* and *trans* subensembles*,* they are limited by the slow timescales of isomerization and force field inaccuracies. NMR spectroscopy can report on ensemble-averaged observables for both the *cis-*proline and *trans-*proline states, but a full atomistic characterization of these conformers is challenging. Given the importance of proline *cis/trans* isomerization for influencing the conformational sampling of disordered proteins, we employed a combination of all-atom MD simulations with enhanced sampling (metadynamics), NMR, and small-angle x-ray scattering (SAXS) to characterize the two subensembles of the ORF6 C-terminal region (ORF6_CTR_) from SARS-CoV-2 corresponding to the proline-57 (P57) *cis* and *trans* states. We performed MD simulations in three distinct force fields: AMBER03ws, AMBER99SB-*disp*, and CHARMM36m, which are all optimized for disordered proteins. Each simulation was run for an accumulated time of 180–220 *μ*s until convergence was reached, as assessed by blocking analysis. A good agreement between the *cis*-P57 populations predicted from metadynamic simulations in AMBER03ws was observed with populations obtained from experimental NMR data. Moreover, we observed good agreement between the radius of gyration predicted from the metadynamic simulations in AMBER03ws and that measured using SAXS. Our findings suggest that both the *cis*-P57 and *trans*-P57 conformations of ORF6_CTR_ are extremely dynamic and that interdisciplinary approaches combining both multiscale computations and experiments offer avenues to explore highly dynamic states that cannot be reliably characterized by either approach in isolation.

## Significance

This study employs MD simulations (with metadynamics), NMR spectroscopy, and SAXS to elucidate the individual *cis-*proline and *trans*-proline conformations of ORF6_CTR_ from SARS-CoV-2. The good agreement on proline *cis*/*trans* populations observed in experiments (NMR) and those calculated from simulations in the AMBER03ws force field (with SAXS reweighting) showcases the efficiency of this interdisciplinary approach, which can be used to characterize highly dynamic disordered protein states, even for very slow processes. Furthermore, our study emphasizes the importance of considering both computational and experimental methodologies to gain a more holistic understanding of highly dynamic proteins. The presented integrative approach sets a precedent for future studies aiming to explore complex and dynamic biological systems with slow transitions such as proline isomerizations.

## Introduction

Intrinsically disordered proteins (IDPs) and disordered regions, which represent at least 30% of the human proteome (1), are particularly common in cancer-associated proteins, with up to 80% containing disordered regions (2), and in viruses, where their coverage ranges from 3 to 55% depending on the viral species (3). Unlike folded proteins, disordered proteins are highly dynamic, and they often exist as an ensemble of diverse heterogeneous conformations that lack a single three-dimensional (3D) structure. Compared with folded proteins, the primary sequences of disordered proteins have a nearly 2-fold increase of proline residues (4), which are well-known to reduce the formation of secondary structure in proteins (5). In particular, proline residues in disordered proteins have been shown to play key roles in regulating protein-protein interactions (6,7), posttranslational modifications (8), and liquid-liquid phase separation (9).

Most peptide bonds within proteins exist almost exclusively in the energetically favorable *trans* conformation. However, for proline residues, the free energy difference between the *cis* and *trans* isomers is lower due to the cyclic structure of this amino acid. Given the high energy barrier to rotation, approximately 84 kJ mol^−1^ (10), proline isomerization is generally a slow process (11), occurring at a rate of 10^−3^–10^−2^ s^−1^ at room temperature, depending on the adjacent residues (12,13). The *cis*-proline population typically ranges between 5 and 10% in disordered proteins (4), but this can vary substantially depending on the length and composition of the amino acid sequence (6,14,15). Consequently, multiple *cis*-proline conformations may be present within polyproline disordered protein ensembles. These ensembles sample a vast conformational space of very slowly exchanging conformers, further increasing their complexity (16).

Molecular dynamics (MD) simulations are often used to characterize the ensemble of disordered proteins as they can resolve individual conformations within an ensemble at atomic resolution, which is a challenge for many experimental techniques. Significant progress has been made over the last decade to optimize force fields for modeling disordered proteins (17,18,19), as well as advances in the integration of MD simulations and experimental data to improve their accuracy (20,21). Despite these advances, sampling the full configurational energy landscape of disordered protein ensembles in all-atom explicit solvent MD simulations is extremely computationally expensive. Proline *cis/trans* isomerization presents an additional challenge due to the slow timescales of this process (12,13), which are generally not accessible in brute-force MD simulations alone (22), even on today’s most powerful computers. However, when suitable collective variables (CVs) can be identified, metadynamics, an enhanced sampling approach, offers an effective method for sampling slow motions (23,24). Indeed, metadynamics has been used to encourage exploration of the full configurational space of disordered proteins (7,25) and proline *cis/trans* isomerization in simulations of dipeptides and folded systems (26,27). In the latter cases, the *ζ* angle (C^α^_*i*–1_, O_*i*–1_, C^δ^_*i*_, C^α^_*i*_*,* where *i* = proline) was employed as one CV for the isomerization and pyramidalization of the amide nitrogen and the *ψ* angle (N_*i*_, C^α^_*i*_, C’_*i*_, N_*i*+1_) was employed as an additional CV to control the amide orientation, which may affect the rate of transition between the *cis-*proline and *trans*-proline conformations. Both CVs are required to enhance proline *cis*/*trans* sampling as they compensate for each other.

NMR spectroscopy is a well-suited experimental technique to characterize ensemble-averaged properties of disordered proteins at atomic resolution under physiological conditions (pH, temperature, and salt concentrations) (28). Furthermore, NMR can uniquely characterize and quantify the populations of *cis-*proline and *trans-*proline conformations. The distinct chemical environments for the two proline isomers, coupled with their slow exchange, can result in the detection of two separate peaks for neighboring residues or the proline itself. NMR has therefore not only been used to characterize the overall ensemble of disordered proteins (29,30), but NMR has also been used extensively to characterize the structural propensities and dynamics of *cis*-proline conformations in disordered proteins (6,14,15,31).

Another experimental technique that can report on the ensembles of disordered proteins in solution is small-angle x-ray scattering (SAXS). This technique can provide coarse structural information relating to a protein’s size and shape. The capability to predict SAXS profiles from atomic coordinates makes it possible to compare conformational ensembles from MD simulations with experimental SAXS data (32). While SAXS measurements offer powerful global information, they report ensemble-averaged states and cannot generally distinguish between the *cis*-proline and *trans*-proline configurations. Complementary approaches, such as NMR, are essential for providing detailed experimental information at the local scale.

Here, we used an integrative approach anchored in all-atom explicit solvent metadynamic simulations to characterize the C-terminal region of open reading frame 6 (ORF6_CTR_) from severe acute respiratory syndrome coronavirus 2 (SARS-CoV-2). This region of ORF6 is predicted to be disordered (Fig. S1, *A* and *B*) and binds to host proteins via an essential methionine residue at position 58 (M58), leading to suppression of the innate immune response (33,34,35). Moreover, this 21-residue peptide contains a single proline residue at position 57 (P57), which may influence its binding to host proteins as this residue is at a preceding position to M58 (33,34). We sampled the conformational space of ORF6_CTR_ using three different force fields, each optimized for disordered proteins: AMBER03ws (a03ws) (17), AMBER99SB-*disp* (a99SB-*disp*) (18), and CHARMM36m (C36m) (19). We employed metadynamics to enhance sampling (23,24), using various local and global CVs, including those on the P57 *ζ* and *ψ* angles (26,27). To reweight and validate resulting conformational ensembles, we compared ensemble-averaged properties from each force field to NMR and SAXS data. Specifically, we employed NMR chemical shifts to report on the local properties and populations of the *cis*-P57 and *trans*-P57 states, NMR diffusion experiments to compare the global properties of both states, and NMR spin-relaxation experiments to probe dynamics. Moreover, SAXS data were used to select the most accurate force field for predicting the ORF6_CTR_ global conformational ensemble. To further refine the conformational ensembles, we updated the statistical reweighting using a Bayesian/maximum entropy (BME) approach (21,36).

By integrating metadynamic simulations, SAXS, and NMR, we can characterize the highly dynamic *cis*-P57 and *trans*-P57 subensembles of ORF6_CTR_. We show that metadynamics with the P57 *ζ* and *ψ* angle CVs enhances sampling of P57 isomerization, and we observe convergence of these two CVs for the a03ws and C36m force fields. We find that a03ws most accurately predicts the *cis*-P57 and *trans*-P57 populations in the ORF6_CTR_. By employing SAXS BME reweighting (21,36) and two independent a03ws runs, we obtain *cis*-P57 populations in the a03ws force field that match those from NMR. NMR diffusion experiments suggest that the *cis*-P57 subensemble is slightly more compact than the *trans*-P57 subensemble, in qualitative agreement with the metadynamic simulation predictions. Furthermore, NMR spin-relaxation experiments and metadynamic simulations indicate that both the *cis*-P57 and the *trans-*P57 conformations of ORF6_CTR_ are extremely dynamic. We anticipate that this interdisciplinary approach can be broadly applied to the many disordered proteins that undergo complex dynamics across varying timescales.

## Materials and methods

### ORF6_CTR_ peptide synthesis

The ORF6_CTR_ with sequence SKSLTENKYSQLDEEQPMEID was initially made by solid-phase peptide synthesis using a MultiSynTech Syro Peptide Synthesiser. Fmoc-Asp(O^t^Bu)-NovaSyn TGT resin and standard Fmoc-amino acids, coupling, and deprotection conditions were used. All residues were double-coupled. The resin was cleaved using a cleavage mixture of TFA/TIPS/H_2_O (95:2.5:2.5) and the peptide was isolated by precipitation from diethyl ether, centrifugation, and lyophilization. A portion (4 mg) of the crude peptide was purified by semipreparative HPLC to give pure ORF6_CTR_ (1.5 mg) (see Fig. S2, *A* and *B* in the supporting material).

### N-Acetylated ORF6_CTR_ peptide synthesis

The unlabeled N-acetylated ORF6_CTR_ peptide (NAc-ORF6_CTR_) with sequence Ac-SKSLTENKYSQLDEEQPMEID was produced synthetically (>96.3% purity) by GenScript (GenScript Biotech UK, Oxford, UK).

### Expression and purification of the isotopically labeled ORF6_CTR_ peptide

The uniformly isotopically labeled ORF6_CTR_ with sequence SKSLTENKYSQLDEEQPMEID was produced by recombinant protein expression with an N-terminal glutathione S-transferase (GST) tag followed by a tobacco etch virus protease cleavage site in the pGEX-6P-1 expression vector (GenScript Biotech UK, Oxford, UK). ORF6_CTR_ was expressed in *Escherichia coli* BL21(DE3) strain. The cells were grown at 37°C in minimal M9 medium containing 1 g/L ^15^NH_4_Cl as the sole nitrogen source and 10 g/L glucose for the ^15^N-labeled ORF6_CTR_ peptide. For the ^13^C,^15^N-labeled ORF6_CTR_ peptide, 1 g/L ^15^NH_4_Cl was used as the sole nitrogen source and 3 g/L ^13^C-glucose as the sole carbon source. Cultures were grown at 37°C with vigorous shaking. Expression was induced at an OD_600_ of 0.5–0.6 by addition of 1 mM isopropyl β-D-thiogalactopyranoside and left shaking for 4 h at 37°C.

The cell pellet was collected by centrifugation and resuspended in lysis buffer containing 50 mM Tris (pH 8.0), 300 mM NaCl, 10 mM β-mercaptoethanol, and 5% glycerol. Prior to lysing the cells via sonication, 0.25% IGEPAL was added to the mixture along with small amounts of DNase, lysozyme, and one protease inhibitor tablet per 50 mL. Next, the cell lysate was added to Glutathione Agarose 4B resin (Protino) and binding was allowed to occur by gently rocking the mixture for 1 h at 4°C. The gravity columns were then washed with the lysis buffer to remove any unbound proteins. ORF6_CTR_ was cleaved from the GST-tag via the addition of tobacco etch virus protease overnight, shaking at 22°C. The next day, the flowthrough containing the cleaved ORF6_CTR_ was concentrated by ultracentrifugation through 1 kDa cutoff centricons (PALL, New York, NY) ultrafiltration membranes. For further purification, size-exclusion chromatography on a Superdex 75 column was carried out in NMR buffer containing 25 mM HEPES (pH 6.9), 150 mM NaCl at 5°C. Fractions containing ORF6_CTR_ were pooled together and concentrated by 1 kDa cutoff centricons (PALL) ultrafiltration membranes. The yield of purified peptide was around 1.4–1.6 mg from 1 L of culture medium.

### NMR spectroscopy

Unless specified otherwise, NMR spectra were collected on uniformly ^15^N-labeled or ^13^C,^15^N-labeled ORF6_CTR_ peptide samples at concentrations of 300 *μ*M and unlabeled NAc-ORF6_CTR_ at concentrations of 400 *μ*M. All peptide samples were prepared in 25 mM HEPES buffer (pH 6.9), 150 mM NaCl, containing 5% D_2_O, 1 mM sodium azide, and 1 mM EDTA. Before recording experiments, ORF6_CTR_ samples were boiled in the NMR tube (sample volume ∼600 *μ*L) at 100°C for ∼3 min to remove proteases. NMR data were recorded at 15°C unless stated otherwise. NMR data were acquired at three different static magnetic fields: 14.1 T (600 MHz) on a Bruker NEO spectrometer with a TXO cryoprobe, 18.8 T (800 MHz) on a Bruker Avance III HD spectrometer equipped with Z-gradient triple-resonance TCI cryoprobe, and 22.3 T (950 MHz) on a Bruker Avance NEO spectrometer equipped with a QCI-F cryoprobe.

Resonance assignments were obtained from a standard suite of double-resonance and triple-resonance experiments at a static magnetic field strength of 14.1 T. Backbone ^15^N relaxation rates, including *R*_1_, *R*_1ρ_, and {^1^H}-^15^N steady-state heteronuclear NOEs (hetNOEs), were measured at two static magnetic field strengths (14.1 and 18.8 T). The backbone ^15^N exchange-free relaxation rates (*R*_dd_) were recorded using a previously described method (37) at a static magnetic field strength of 14.1 T. Diffusion ordered spectroscopy (DOSY) experiments were measured using a pseudo-3D ^1^H-^15^N heteronuclear single quantum coherence (HSQC)-type experiment at a static magnetic field strength of 22.3 T. Additional information regarding the NMR experiments can be found in the supporting material.

### NMR data analysis

All NMR spectra were processed using NMRPipe (38), and analyzed with NMRFAM-SPARKY software (39) and FuDA (37). To analyze residual secondary structure for the *cis*-P57 and *trans*-P57 configurations, we used the secondary structure propensity score algorithm (40), with the ORF6_CTR_^1^H^α^, ^13^C^α^, and ^13^C^β^ chemical shifts as input. *R*_1_ and *R*_1ρ_ rate-constants were calculated by fitting the intensity profiles to monoexponential decay functions with FuDA (37). The {^1^H}-^15^N hetNOEs were calculated as the ratios of the peak intensities in the saturated and reference subspectra. The transverse relaxation rate constants (*R*_2_) were calculated from the *R*_1_ and *R*_1ρ_ relaxation rates (see Eqs. S1 and S2 in the supporting material). Peak intensities of the diffusion NMR data were obtained with FuDA (37) and diffusion coefficients were calculated (see Eq. S3 in the supporting material).

### SAXS measurements

The NAc-ORF6_CTR_ was dissolved in 25 mM HEPES (pH 6.9), 150 mM NaCl and centrifuged at 5600 × *g* for 10 min at 4°C to give a final concentration of 2 mg/mL (800 *μ*M). The SAXS data were obtained on Instrument B21 at Diamond Light Source (Didcot, UK) (41). Measurements were recorded at 37°C. Data sets of 26 frames with a frame exposure time of 1 s each were acquired. ScÅtter IV (42) was used for buffer subtraction and data reduction, in which the 26 frames were averaged. The Guinier peak analysis function in ScÅtter IV was used to calculate the radius of gyration (*R*_g_). Furthermore, we rescaled the error bars for the SAXS intensities by a factor estimated through the Bayesian indirect Fourier transform using the BioXTAS Raw software (43).

### MD simulations

All-atom metadynamic simulations were performed using GROMACS 2021.2 (44) patched with the open-source, community-developed PLUMED library version 2.7.1 (45). Simulations were setup using three different force fields and their corresponding water models: a03ws force field (17) with the TIP4P/2005 water model (46), a99SB-*disp* (18) with the TIP4P-D water model (47), and C36m (19) with the CHARMM-modified TIP3P water model (48). ORF6_CTR_ was N-acetylated for the simulations. The initial starting structure for NAc-ORF6_CTR_ was prepared as a linear peptide using PyMOL (49). Topology and coordinate files were then generated for the peptide using the heavy hydrogen flag for hydrogen mass repartitioning (50). The system was then solvated in a rhombic dodecahedron box with an initial volume of 775 nm^3^. Ions were added to neutralize the charge of the system and to maintain a concentration of 150 mM NaCl to match experiments. The solvated system was subjected to an energy minimization simulation using the steepest descent method with a target maximum force of 2,000 kJ mol^−1^ nm^−1^. The extended structure was collapsed by running a high-temperature simulation at 600 K for 20 ns in the canonical (NVT) ensemble with a 2 fs time step. To ensure a sufficiently large box size, the *R*_g_ was calculated for each frame in the 600 K NVT trajectory. From the 95th percentile of the *R*_g_ distribution, a collapsed peptide starting structure was randomly selected. This was repeated for each force field.

The collapsed peptide structure was then resolvated and neutralized at a concentration of 150 mM NaCl in a new rhombic dodecahedron box with volumes between 290 and 380 nm^3^ and 9,600–12,200 explicit water molecules, depending on the force field. Each force field system was subjected to the energy minimization simulation by steepest descent with a target maximum force of 2,000 kJ mol^−1^ nm^−1^. All subsequent simulations employed either a 4 or 5 fs time step to enhance computational efficiency. A 20 ns simulation at 600 K in the NVT ensemble was performed to obtain 128 diverse starting structures. These 128 starting structures were taken at even intervals after 5 ns of equilibration time. Thermalization was implemented for 1,250 ps at 310 K in the canonical (NVT) ensemble using the Bussi-Donadio-Parrinello thermostat (51). Then the density of each system was equilibrated for 50 ns at 310 K in the isothermal-isobaric (NPT) ensemble using the Parrinello-Rahman barostat (52).

Production runs were executed in the NPT ensemble with a target pressure of 1 bar and temperature of 310 K, employing the Parrinello-Rahman barostat (52), and using a 5 fs time step (50). LINCS constraints on all bonds were used (53). A Verlet list cutoff scheme was used for the nonbonded interactions. The van der Waals and Coulomb interactions were cut off at 1.2 nm for all a03ws and a99SB-*disp* simulations and at 0.95 nm for C36m. Long-range electrostatic effects were treated with the particle-mesh Ewald method (54). Metadynamics was performed with the parallel-bias, well-tempered, and multiple-walkers protocols using a Gaussian deposition stride set to 2.5 ps, an initial height of 1.2 kJ mol^−1^, and a bias factor of 30 for 128 replicas (23,24,25). Nine CVs were selected to enhance conformational sampling (see Eqs. S4–S8; Table S1). Metadynamic simulations were run for an accumulated time of 192 *μ*s (a03ws run 1 and a03ws run 2), 181 *μ*s (a99SB-*disp*), and 222 *μ*s (C36m) until convergence was reached as assessed by blocking analysis for every CV (see Figs. S3, *A*–*C* and S4; Eq. S9) (32,55).

### Structural ensemble analysis

Statistical weights were calculated for each force field at the end of the simulation (see Eq. S10). Analysis was conducted using the open-source package MDTraj (56). Chemical shift predictions were back-calculated from the simulations at each time step using CamShift (57). SAXS intensity curves were calculated for each structure using Pepsi-SAXS (58) and we followed a previously reported method to perform the fitting (36). We used the BME software to update the metadynamics weights to better match experimental SAXS data (21).

## Results

### Sampling slowly exchanging proline *cis/trans* conformers with metadynamic simulations

To characterize the structural ensemble of ORF6_CTR_, all-atom explicit solvent MD simulations were set up with enhanced sampling provided by metadynamics (23,24). Given the sensitivity of simulations of disordered proteins to the force field used (59), we employed three diverse parameter sets (force fields) optimized for disordered proteins, capable of sampling both folded and disordered regions, or primarily extended conformations. These included the force fields a03ws (17), a99SB-*disp* (18), and C36m (19). The ORF6_CTR_ peptide was N-acetylated (NAc-ORF6_CTR_) in the simulations to ensure a neutral charge at the N-terminus and to better match the peptide in its native context.

**Figure 1:**
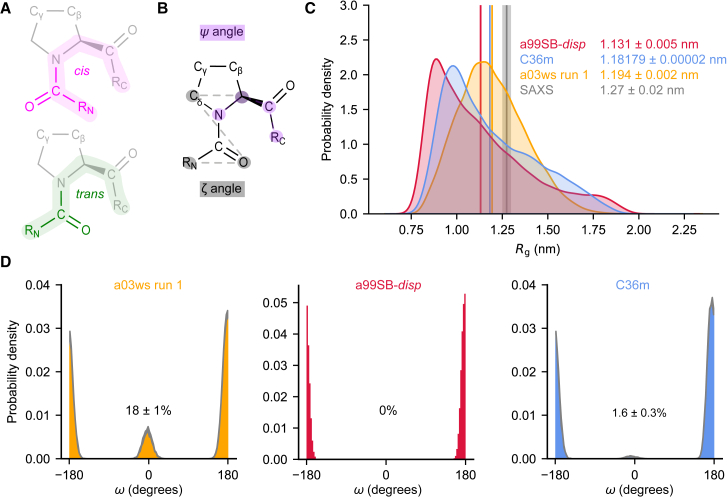
All three force fields predict NAc-ORF6_CTR_ is disordered but only the a03ws and C36m force fields predict that NAc-ORF6_CTR_ undergoes proline *cis/trans* isomerization. (*A*) *cis*-proline (*pink*) and *trans*-proline (*green*) conformations with the positions of C^γ^ and C^β^ indicated. (*B*) Definition of the *ζ* (*gray*) and *ψ* (*purple*) dihedral angles used as CVs to enhance proline *cis/trans* isomerization. (*C*) *R*_g_ probability distributions were calculated using kernel density estimates to compare the a03ws run 1 (*orange*), a99SB-*disp* (*red*), and C36m (*blue*) ensembles. Ensemble-averaged *R*_g_ are shown for each system. The associated error represents the standard deviation between the first and second halves of the analyzed trajectories. The experimental SAXS data and error (standard deviation from the Guinier analysis) are shown in gray. (*D*) Probability distributions for the *ω* angle about the Q56-P57 peptide bond in the a03ws run 1 (*orange*), a99SB-*disp* (*red*), and C36m (*blue*) ensembles. The thickness of the gray distribution represents the uncertainty. The *cis*-P57 populations are displayed for each system and the error represents the standard deviation between the first and second halves of the analyzed trajectories.

To obtain a good sampling of the highly heterogeneous free energy surface (FES) adopted by the expected disordered peptide (Fig. S1, *A* and *B*), we selected a variety of metadynamics CVs to explore both local and global properties of NAc-ORF6_CTR_. These CVs included total α-helical content, total β-sheet content, the radius of gyration (*R*_g_), the number of salt bridges, the distance between the C^α^ of the first and last residue (end-to-end distance), the correlation between consecutive *ψ* dihedral angles (dihedral correlation), and the number of contacts between hydrophobic residues (25). To enhance proline *cis/trans* isomerization (Fig. 1 *A*), we also implemented two CVs on the P57 improper *ζ* dihedral angle and the P57 *ψ* dihedral angle (Fig. 1 *B*) (see the supporting material for more information on the CVs) (26,27). Each CV for each force field was assessed for convergence by blocking analysis (see Fig. S3, *A*–*C*; Eq. S9) (32,55). We observed that the FES standard-error plateaus at a constant value for the CVs listed above in all three force fields, except for the P57 *ζ* improper dihedral angle CV in the a99SB-*disp* force field.

For the a03ws and C36m force fields, convergence was observed for the P57 *ζ* improper dihedral angle (Fig. S3, *A* and *C*). This, combined with convergence of the *ψ* dihedral angle, suggests that P57 *cis/trans* isomerization was converged despite the very slow timescale generally observed for proline isomerization, typically 10–10^3^ s at room temperature (12,13). Sampling of the *cis*-P57 isomer was not observed in the simulation with the a99SB-*disp* force field (Fig. S3 *B*). This is potentially due to the exclusion of glycine and proline residues from torsion optimizations, along with the enhanced Lennard-Jones pair override (18). New and improved force fields, such as DES-Amber (60), have further advanced upon this and may be better suited for proteins with both ordered and disordered regions or for investigating a disordered protein in complex with a folded protein.

To assess the sampling of secondary structures, we calculated ensemble-averaged C^α^ minimum distance contact maps for all three force fields, which showed low probabilities of α-helix and β-sheet content (Fig. S5, *A–C*), and thus a lack of secondary structure. This result is consistent with predictions from s2D (Fig. S1 *A*), a protein secondary structure propensity predictor trained on solution-based NMR data (61), and the AlphaFold2 pLDDT score (62), in which low confidence scores have been found to correlate with disordered regions (Fig. S1 *B*) (63).

### Assessing the conformational ensembles using experimental data

Histograms of the *R*_g_, calculated from the ensembles obtained with metadynamic simulations, show that NAc-ORF6_CTR_ adopts a continuum of states in all three force fields, ranging from collapsed to extended, suggesting that NAc-ORF6_CTR_ exists as an ensemble of disordered conformations (Fig. 1 *C*), in agreement with the lack of stable secondary structure (Fig. S5, *A–C*). It is well established that various force fields for disordered proteins can predict dramatically different features for the same disordered protein sequence, particularly in terms of ensemble-averaged properties such as *R*_g_ values (59). The three force fields used herein predict slightly different *R*_g_ values (a03ws run 1: 1.194 ± 0.002 nm, a99SB-*disp*: 1.131 ± 0.005 nm, and C36m: 1.18179 ± 0.00002 nm; Fig. 1 *C*). To assess which of the simulations, if any, was the most in agreement with experimental measurements, we performed SAXS experiments on NAc-ORF6_CTR_ and determined an ensemble-averaged *R*_g_ of 1.27 ± 0.02 nm. Notably, a03ws and C36m performed only marginally better than the coarse-grained force field CALVADOS, which predicts an *R*_g_ of 1.180 ± 0.007 nm (64,65,66). Furthermore, the coarse-grained disordered region ensemble predictor ALBATROSS (67) predicts an *R*_g_ of 1.29 nm, which is closer in agreement with the experimental *R*_g_ than any of the all-atom models tested here. Both coarse-grained force field predictors specifically used the *R*_g_ parameter for optimization (66,67).

In contrast to SAXS data, which report on global properties, NMR chemical shifts report on local dihedral angles, ring current shifts, and electrostatics (68), and are regularly used to improve and assess the accuracy of simulation ensembles because they are sensitive to secondary structure (21,25). To this end, we recorded and assigned 2D ^1^H–^1^H total correlation spectroscopy (TOCSY) and ^1^H-^13^C HSQC spectra of unlabeled NAc-ORF6_CTR_ to obtain ^13^C^α^, ^13^C^β^, ^1^H^α^, and ^1^H^N^ chemical shifts. Using CamShift (57), we back-calculated chemical shifts for all nonproline residues in our metadynamics ensembles for all three force fields. All experimentally determined chemical shifts were within the CamShift error (Fig. S6, *A–C*). To probe secondary structure in the Nac-ORF6_CTR_ we recorded a 2D ^1^H–^1^H TOCSY temperature titration at eight temperature values between 5 and 37°C. The linear temperature coefficients of the ^1^H^N^ chemical shifts are indicative of the hydrogen bonding state of individual amides (69). For example, at higher temperatures hydrogen bonds are weakened, which causes the relative upfield shifting of the ^1^H^N^ chemical shift. All residues in the Nac-ORF6_CTR_ have temperature coefficients more negative than −4.5 ppb K^−1^ (Fig. S7), suggesting that none of the ^1^H^N^ are involved in a long-lived intramolecular hydrogen bond. This indicates that there is no substantial secondary structure in Nac-ORF6_CTR_, in agreement with the metadynamic simulations.

### Solution-state NMR confirms proline *cis/trans* isomerization

Like force-field-dependent variations predicted in the *R*_g_ distributions, the *cis*-P57 population predictions also vary dramatically between different force fields (Fig. 1 *D*). Our analysis suggests *cis*-P57 populations of 18 ± 1, 0, and 1.6 ± 0.3% using the a03ws run 1, a99SB-*disp*, and C36m force fields, respectively. To assess the accuracy of these *cis*-P57 populations, we recombinantly expressed and purified uniformly ^15^N-labeled and ^13^C, ^15^N-labeled ORF6_CTR_ for NMR experiments (see materials and methods). The production of isotopically labeled ORF6_CTR_ in *E. coli* required a GST-tag, making it extremely difficult to N-acetylate the system. While N-acetylation has been shown to decrease the *cis*-proline population in small tetrapeptide systems, the effect of N-acetylation on *cis*-proline sampling is reduced upon increasing the length of the peptide (14). As the N-acetylation in ORF6_CTR_ would occur at residue S41, we anticipate that potential *cis/trans* isomerization at residue P57, separated by 16 residues from the acetylation site, is unlikely to have its chemical environment influenced by the acetyl group (see below).

**Figure 2:**
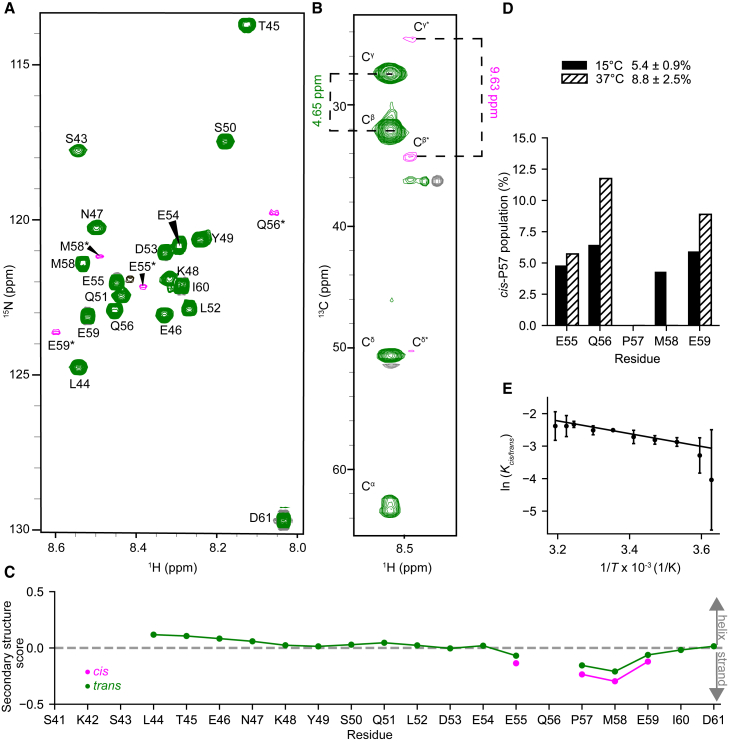
NMR verifies the disordered ORF6_CTR_ undergoes proline *cis/trans* isomerization. (*A*) 2D ^1^H-^15^N HSQC spectrum of 300 *μ*M ^15^N-labeled ORF6_CTR_ measured at 15°C, pH 6.9, and 14.1 T. Residues colored in green originate from the *trans-*P57 conformational ensemble, while residues colored in pink and labeled with an asterisk (^∗^) report on the *cis*-P57 conformation. The black peak is unassigned. (*B*) 2D ^1^H-^13^C strip taken from the 3D CC(CO)NH spectrum of the 300 *μ*M ^13^C,^15^N-labeled ORF6_CTR_ at the ^15^N chemical shift of M58^∗^. (*C*) The ORF6_CTR_^13^C^α^, ^13^C^β^, and ^1^H^α^ chemical shifts were used to calculate the secondary structure propensity scores for both the *cis*-P57 and *trans*-P57 subensembles. A positive value indicates α-helical propensity, a negative value indicates a β-strand propensity, whereas a value near zero indicates random coil. (*D*) The *cis*-P57 populations at 15°C (*solid fill*) and 37°C (*dashed fill*) are shown for each well-resolved *cis*-P57 and *trans-*P57 peak in the 2D ^1^H-^15^N HSQC spectrum. The integrated peak volume was used to calculate the mean *cis*-P57 population and standard deviation across residues E55, Q56, and E59. (*E*) The van ’t Hoff analysis of P57 *cis/trans* isomerization determined from the integrated peak volume. The mean natural logarithm of the equilibrium constant for proline *cis*/*trans* isomerization about the Q56-P57 peptide bond as a function of temperature was calculated using residues E55, Q56, and E59. Error bars represent the standard deviation across the three residues used in the analysis. The van ’t Hoff linear fit yielded a *cis*-P57 population of 10 ± 2% at 37°C.

2D ^1^H-^15^N HSQC spectra show sharp resonances with a limited chemical shift dispersion in the ^1^H^N^ dimension, suggesting that these resonances arise from disordered residues (Fig. 2 *A*). This finding agrees with spectra of the unlabeled, N-acetylated peptide (Fig. S8), and with the secondary structure predictions (Fig. S1, *A* and *B*). The chemical shifts in the ORF6_CTR_ are very similar to those obtained from the NAc-ORF6_CTR_ (see Table S2; Eq. S11). The a03ws and C36m simulations discussed above predict that the ORF6_CTR_ conformational ensemble undergoes proline *cis/trans* isomerization about the Q56-P57 peptide bond. Indeed, in the 2D ^1^H-^15^N HSQC spectrum, two distinct signals with different signal intensities are observed for residues in proximity to P57 (Fig. 2 *A*).

To verify that the additional set of minor peaks corresponds to residues in the *cis*-P57 conformational ensemble, we analyzed the ^13^C side-chain chemical shifts of P57 using a 3D CC(CO)NH experiment (Fig. 2 *B*)(70). The difference in chemical shifts between ^13^C^β^ and ^13^C^γ^ is highly diagnostic of a *cis-*proline (∼9.5 ppm) or a *trans*-proline (∼4.5 ppm) peptide bond conformation (31,71). Similarly, peaks from the *cis*-P57 ensemble were detected for residues E55, Q56, M58, and E59. Most *cis-*P57 peaks are relatively close to their corresponding *trans-*P57 peak. For Q56, the residue preceding P57, the change in the ^15^N chemical shift between the *cis* and *trans* states is ∼3 ppm. A large change in chemical shift between *cis-*proline and *trans*-proline configurations is not uncommon for peaks of residues preceding the proline undergoing isomerization (71). Thus, NMR provides evidence of a *cis*-P57 conformation, while also enabling the characterization of the properties of both the proline *cis* and *trans* conformational subensembles. Initially, to characterize the secondary structure content of the *cis-*P57 and *trans-*P57 subensembles, we calculated the secondary structure propensity scores for the two configurations (40). These scores indicate that both conformations are disordered (Fig. 2 *C*), although there is a slight increase in β-strand propensity near the C-terminus for the *cis-*P57 subensemble.

In the slow-exchange regime, NMR peak intensities are proportional to the concentration of the species giving rise to the observed peaks (72). The population of the *cis-*P57 and *trans-*P57 conformations can thus be determined, assuming slow exchange and similar dynamics for the two conformations. The NMR experiments described above were recorded at 15°C to reduce signal loss due to backbone amide proton exchange with the solvent. The *cis*-P57 and *trans*-P57 peak intensities at 15°C were calculated for residues E55, Q56, M58, and E59 using the integrated peak volume or the peak height (Figs. 2 *D* and S9 *A*) (31). To enhance the *cis*-P57 population, study the system at a biologically relevant temperature, and match our metadynamic simulations, the *cis*-P57 and *trans*-P57 integrated peak volumes and peak heights were also analyzed at 37°C. Residue M58 was excluded from the analysis at 37°C since the two proline isomer peaks overlap at higher temperatures. The integrated peak volume analysis yielded a *cis*-P57 population of 5.4 ± 0.9% at 15°C and 8.8 ± 2.5% at 37°C (Fig. 2 *D*), which agrees most closely with the free energy difference between the *cis*-P57 and *trans*-P57 conformations in the a03ws run 1 metadynamic simulation.

To investigate the enthalpic and entropic factors influencing the formation of the *cis*-P57 state, we measured the equilibrium constant K = [*cis*]/[*trans*] for residues E55, Q56, and E59 at 10 temperature values between 2.5 and 40°C. From the van ’t Hoff analysis, using integrated peak volumes, we observed that the *cis*-P57 conformation is enthalpically disfavored (Δ*Η* = 16 ± 2 kJ mol^−1^) but entropically favored (Δ*S* = 34 ± 7 J K^−1^ mol^−1^) over the *trans*-P57 conformation (Fig. 2 *E*). Similar results were obtained using peak heights, where Δ*Η* = 14 ± 1 kJ mol^−1^ and Δ*S* = 25 ± 3 J K^−1^ mol^−1^ (Fig. S9 *B*). The *cis*-P57 population is consistent with results reported in the literature for other disordered protein systems (31) and is a reasonable population given that the Q56 residue, preceding P57 in ORF6_CTR_, is neither favorable nor unfavorable for proline isomerization (71).

### Refining the metadynamics ensembles using Bayesian/maximum entropy

Given that the a03ws force field (a03ws run 1) produced an ensemble that agreed best with the experimental *cis*-P57 populations and SAXS data, we repeated the a03ws simulation (a03ws run 2) to assess reproducibility. To do this, and to avoid positive bias for the *cis*-P57 configuration, we randomly selected 128 frames only from the *trans*-P57 configuration within the initial a03ws run 1 ensemble. We then repeated the metadynamic simulation until convergence was reached at an accumulated simulation time of 192 *μ*s (Fig. S4). The a03ws run 2 conformational ensemble was again consistent with the NMR data (Fig. S10), gave a *cis*-P57 population of 13 ± 1% (Fig. S11 *A*), and agreed with the characterizations from a03ws run 1 (Figs. 1 *C*, S5 *A*, and S11, *B* and *C*). The robust sampling of the *cis*-P57 conformation, even when using starting structures with 100% *trans*-P57, and the consistency with both SAXS and chemical shift data from two separate metadynamic simulations, indicate that the a03ws force field produces an NAc-ORF6_CTR_ ensemble that is both robust and in agreement with the experimental data for this system.

**Figure 3:**
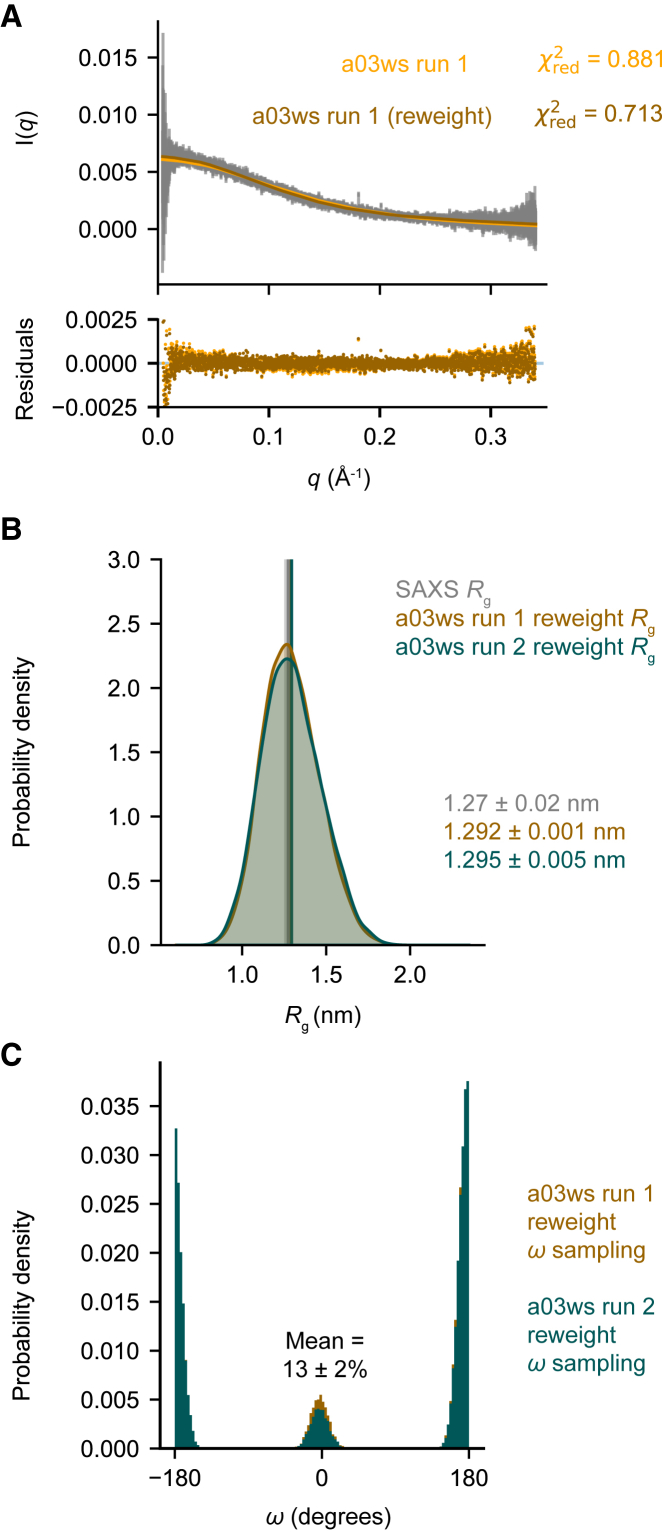
The a03ws force field most accurately predicts the disordered NAc-ORF6_CTR_ ensemble and P57 *cis/trans* populations after SAXS BME reweighting. (*A*) The calculated SAXS intensities from the a03ws run 1 metadynamic simulation (*orange*) and the SAXS BME reweighted simulation (*brown*) compared with the experimental SAXS intensities (*gray*), and the error associated with each intensity (*gray*). (*B*) *R*_g_ probability distributions were calculated using kernel density estimates to compare the SAXS BME reweighted a03ws run 1 ensemble (*brown*) and the SAXS BME reweighted a03ws run 2 ensemble (*teal*). The associated error represents the standard deviation between the first and second halves of the analyzed trajectories. The experimental SAXS data and error (standard deviation from the Guinier analysis) are shown in gray. (*C*) Probability distributions for the *ω* angle about the Q56-P57 peptide bond in the SAXS BME reweighted a03ws run 1 ensemble (*brown*) and the SAXS BME reweighted a03ws run 2 ensemble (*teal*). The two reweighted independent a03ws ensembles were used to calculate the mean *cis*-P57 population and standard deviation. Additional plots for the SAXS BME reweighted a03ws run 2 and C36m ensembles can be found in the supporting material.

To further refine the conformational ensemble with minimal perturbation, we used BME reweighting with the SAXS data for the two independent a03ws ensembles and the C36m ensemble (21,36). While all three ensembles were already in quite good agreement with the SAXS data (Figs. 3 *A* and S12 *A*), we were interested in exploring whether reweighting using SAXS data could improve the *cis-*P57 population predictions. As expected, reweighting resulted in a more extended ensemble-averaged *R*_g_ for the a03ws run 1 (1.292 ± 0.001 nm) and the a03ws run 2 (1.295 ± 0.005 nm) ensembles (Fig. 3 *B*), indicating that the reweighting was successful. Reweighting also increased the ensemble-averaged *R*_g_ of the C36m ensemble (1.299 ± 0.003 nm) and shifted the C36m *R*_g_ distribution to match the a03ws distributions (Fig. S12 *B*–*D*).

Across all three systems, we observed that SAXS BME reweighting led to a reduction in the *cis*-P57 populations (Figs. 3 *C* and S12 *E*). Although reweighting improved the consistency between predicted *cis*-P57 populations in both a03ws ensembles and the NMR data, it did not improve the prediction in the C36m ensemble. This discrepancy underscores the challenge of using BME reweighting to improve accuracy when experimental data are minimally informative on the desired observable, as in this case, where SAXS does not distinguish the *cis-*proline and *trans-*proline conformers. Using experimental data that are informative on both isomeric states could potentially improve the accuracy of the *cis*-proline population predictions. The reweighted ensembles were also compared with the chemical shift data and no significant changes were observed (Fig. S13, *A*–*C*). Again, this indicates that the chemical shifts alone are not sufficient for reweighting the ensembles, as changes to the global conformation have limited influence on this parameter. Taking the two SAXS BME reweighted a03ws ensembles we calculated a mean *cis*-P57 population of 13 ± 2%, which is in good agreement with the population derived from NMR (∼10 ± 2%).

### ORF6_CTR_ is highly dynamic in both proline configurations

**Figure 4:**
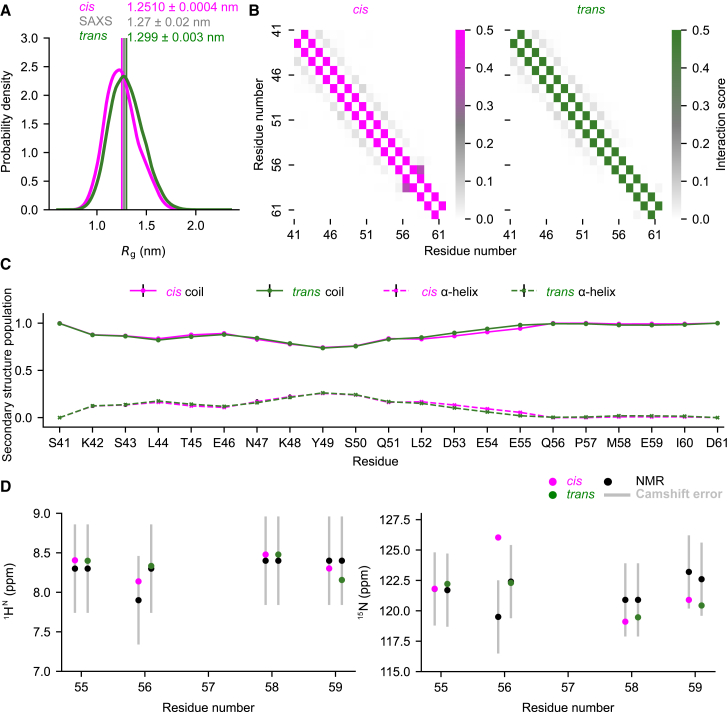
NAc-ORF6_CTR_*cis-*P57 and *trans*-P57 subensembles predicted by the a03ws run 1 SAXS BME reweighted metadynamic simulation have very similar profiles. *(A) R*_g_ probability distributions were calculated using kernel density estimates to compare the *cis*-P57 (*pink*) and *trans*-P57 (*green*) conformational subensembles. The associated error represents the standard deviation between the first and second halves of the analyzed trajectory. The experimental SAXS data and error (standard deviation from the Guinier analysis) are shown in gray. (*B*) Ensemble-averaged C^α^ minimum distance contact maps for the *cis*-P57 (*pink*) and *trans*-P57 (*green*) conformations. Color intensity represents the probability of a contact between residues, with white indicating no contact, and increasing color intensity suggesting higher contact probabilities. (*C*) Secondary structure populations for all residues in the *cis*-P57 (*pink*) and the *trans*-P57 (*green*) conformations based on SAXS BME statistical weights. Coil populations are represented by solid lines and α-helical populations by dashed lines. β-strand represents less than 0.5% of the population for each residue so was not included. Error bars (*black*) represent the standard deviation between the first and second halves of the analyzed trajectory. (*D*) Consistency of the ^1^H^N^ and ^15^N *cis*-P57 and *trans*-P57 ORF6_CTR_ experimental chemical shifts with the predicted chemical shifts from the reweighted NAc-ORF6_CTR_ a03ws run 1 ensemble. The error in CamShift (*silver*) is shown. The standard deviation for predicted chemical shifts between the first and second halves of the analyzed trajectory were also plotted but are too small to see. See the supporting material for the a03ws run 2 and C36m analysis.

Using the reweighted metadynamics ensembles, we next sought to elucidate the structural differences between the *cis*-P57 and *trans*-P57 states. We observed that both conformations have similar *R*_g_ distributions (Fig. 4 *A*), with the *cis-*P57 state exhibiting a slightly smaller mean conformation (1.2510 ± 0.0004 nm) compared with the *trans*-P57 state (1.299 ± 0.003 nm). We then calculated ensemble-averaged C^α^ minimum distance contact maps for all 21 residues (Fig. 4 *B*) and the secondary structure populations for both P57 states (Fig. 4 *C*). Both analyses suggest an overall lack of stable secondary structure for the *cis*-P57 and the *trans*-P57 subensembles. Within the N-terminal region of ORF6_CTR_, the secondary structure propensity scores calculated from NMR chemical shifts and the secondary structure populations from the metadynamic simulations indicate the sampling of very transient α-helical structures (Figs. 2 *C*, 4 *C*, S14 *A–C*, and S15 *A–C*). Whereas the chemical shifts near the P57 residue indicate a slight increase in β-strand (Fig. 2 *C*). These near-miniscule changes in secondary structure were not predicted by the metadynamic simulations ( Figs. 4 *C*, S14 *C*, and S15 *C*).

We also wondered whether the metadynamic simulations could accurately predict both the *cis*-P57 and *trans*-P57 chemical shifts using CamShift (57). Thus, we compared ^1^H^N^ and ^15^N chemical shifts for residues E55, Q56, M58, and E59 with those predicted by CamShift (Figs. 4 *D*, S14 *D*, and S15 *D*). The errors in CamShift are generally greater than the chemical shift differences between *cis*-P57 and *trans*-P57 states, except for the backbone nitrogen for residue Q56, which precedes P57. For this experimental observable, CamShift accurately predicts the value for the *trans*-P57 state, but inaccurately predicts that of the *cis*-P57 state (Figs. 4 *D*, S14 *D*, and S15 *D*). As more data for proline isomerization of disordered proteins becomes readily available, we anticipate that force field parameters for this important state will improve, as well as the prediction of chemical shifts for *cis*-proline states.

To qualitatively compare the global features of the *cis*-P57 and *trans*-P57 ORF6_CTR_ subensembles predicted by the metadynamic simulations to experiments, we recorded ^1^H-^15^N-DOSY-HSQC experiments at a magnetic field strength of 22.3 T. The P57 *cis*/*trans* isomerization exchange rate is slow compared with the diffusion timescales (Δ = 200 ms), and slow compared with the longitudinal relaxation (*k*_ex_ ≪ *R*_1_), making it possible to measure distinct diffusion rates for the *cis*-P57 and *trans*-P57 subensembles. We determined the average diffusion coefficients for the *cis*-P57 and *trans*-P57 conformations using data from residues Q56, M58, and E59. The diffusion measurements for the ^15^N-labeled ORF6_CTR_ at pH 6.9 and 15°C revealed a diffusion coefficient of (2.52 ± 0.09) × 10^−10^ m^2^ s^−1^ for the *cis*-P57 conformation and (2.34 ± 0.05) × 10^−10^ m^2^ s^−1^ for the *trans*-P57 conformation. The observed faster diffusion coefficient for the *cis*-P57 subensemble suggests a slightly more compact conformation compared with the *trans*-P57, with statistical significance of 98% (determined via a one-tailed Welch’s *t*-test, df = 4, *t* = 3.179, *p* = 0.042). This finding is in qualitative agreement with our SAXS BME reweighted metadynamic simulations, which predict that the ORF6_CTR_ *cis*-P57 subensemble is slightly more compact than the *trans*-P57 subensemble in the two independent a03ws simulations and the C36m simulation (Figs. 4 *A*, S14 *A*, and S15 *A*). However, small discrepancies are observed when comparing the relative differences in the predicted ensemble-averaged *R*_g_ values between the *cis*-P57 and *trans*-P57 subensembles with their corresponding NMR diffusion coefficients, highlighting the accuracy limitations of all three metadynamic simulations.

**Figure 5:**
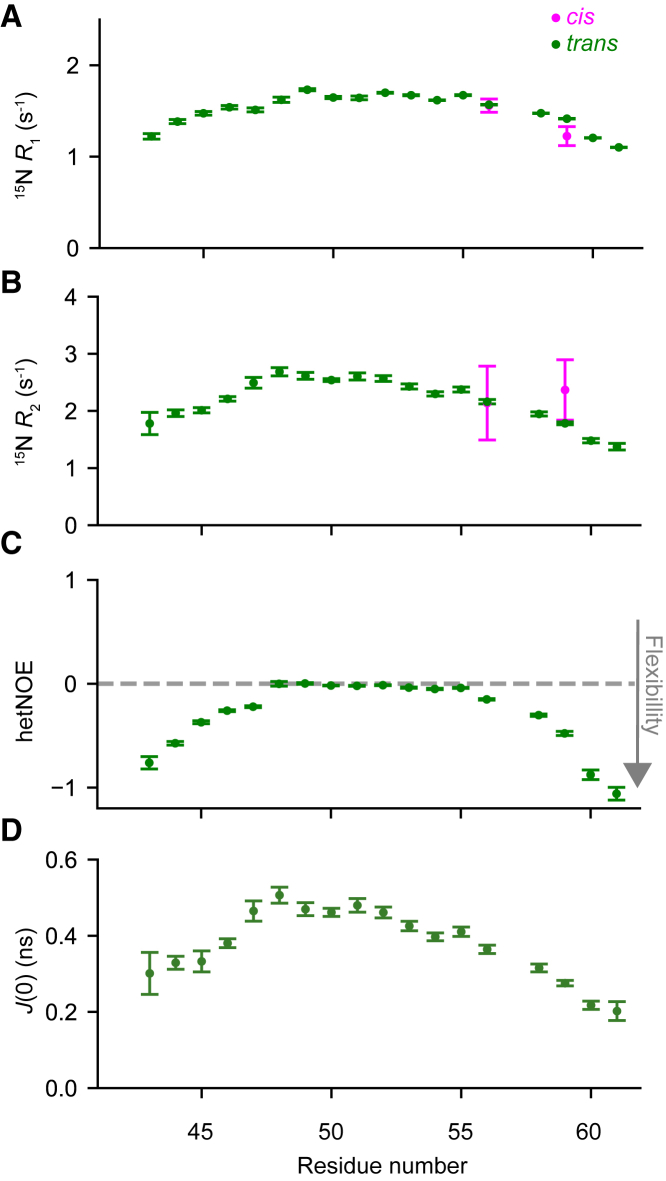
ORF6_CTR_ is very dynamic in both the *trans*-P57 and *cis*-P57 conformations. (*A*) ^15^N longitudinal relaxation rates (*R*_1_), (*B*) ^15^N transverse relaxation rates (*R*_2_), (*C*) {^1^H}-^15^N steady-state hetNOEs, and (*D*) *J*(0) values of 300 *μ*M ^15^N-labeled ORF6_CTR_ at 15°C and pH 6.9. The backbone motions are shown for all peaks corresponding to the *trans*-P57 conformation (*green*), and only for the well-resolved peaks with a measurable intensity corresponding to the *cis*-P57 (*pink*) conformation. Error bars represent the fitting of the NMR data in (*A*) and (*C*). The error bars for (*B*) were calculated by error propagation. The error bars for (*D*) represent the standard deviations for each residue, which were calculated using Monte Carlo uncertainty propagation. All experiments were recorded at a static magnetic field strength of 14.1 T.

To characterize the dynamics of the ORF6_CTR_ experimentally, we recorded the standard set of backbone heteronuclear ^1^H-^15^N relaxation experiments at 15°C and at two different static magnetic field strengths (14.1 and 18.8 T) (73). ^15^N *R*_2_ rates were determined from the corresponding ^15^N *R*_1ρ_ rates, to minimize the effect of off-resonance effects and potential microsecond exchange dynamics (see Eqs. S1 and S2) (74). The ^15^N *R*_1_ and R_2_ relaxation rates report on protein motions occurring at timescales faster than the effective rotational correlation time, which is usually on the nanosecond timescale for disordered proteins. The rates obtained for the ORF6_CTR_ *cis*-P57 and *trans*-P57 subensembles are very similar, and both show the expected bell shape for a random coil disordered state (Figs. 5, *A* and *B* and S16, *A* and *B*) (31,75).

The {^1^H}-^15^N hetNOEs measured for ORF6_CTR_ at a static magnetic field strengths of 14.1 T were mostly near 0 in the central region and decreased to −1 at the N- and C-termini, which is indicative of picosecond dynamics associated with a disordered protein (Fig. 5 *C*) (73). At higher static magnetic field strengths (18.8 T), the values of the steady-state hetNOEs increase relatively, suggesting the absence of slow motions in ORF6_CTR_ (Fig. S16 *C*). We also evaluated the spectral density function, *J*(*ω*), at zero frequency, *J*(0), on a per-residue basis by reduced spectral density mapping (Fig. 5 *D*) (37). This analysis indicated that both P57 subensembles are highly dynamic on the nanosecond timescale and there are no substantial changes in secondary structure propensity between the two ORF6_CTR_ P57 states.

Transverse ^15^N relaxation rates (*R*_2_) also report on the potential chemical exchange contribution (*R*_ex_) due to dynamics occurring on the microsecond to millisecond timescale. One way of distinguishing between the contributions to the ^15^N *R*_2_ rates arising from picosecond to nanosecond dynamics and those associated with slower microsecond to millisecond dynamics is through the use of the ^15^N exchange-free relaxation rate (*R*_dd_) (37). Thus, we determined the *R*_dd_ rates at a static magnetic field strength of 14.1 T (Fig. S17 *A*). We did not detect any exchange occurring on the microsecond to millisecond timescale (Fig. S17, *B* and *C*), suggesting minimal long-range contacts and/or stabilization of secondary structures. This is consistent with the lack of secondary structure observed for both P57 ORF6_CTR_ subensembles from the secondary structure propensity scores (Fig. 2 *C*).

## Discussion

Significant progress has been made over the last decade to accurately characterize the conformational ensembles of disordered proteins with MD simulations and experimental techniques. The extensive amounts of data generated by MD simulations, alongside the capabilities of deep-learning, have now made it possible to predict some overall properties of disordered proteins from just the amino acid sequence alone (66,67). Using deep-learning techniques, global features such as the ensemble-averaged *R*_g_ can be determined within a couple of minutes. This approach achieves an accuracy that matches experimental data and surpasses the accuracy of all-atom explicit solvent MD simulations accumulated over hundreds of microseconds. While these coarse-grained deep-learning approaches have the potential to accurately characterize many global features of disordered proteins, atomic resolution is currently required to characterize local features, such as proline *cis* and *trans* configurations. Proline *cis*/*trans* isomerization, which in certain cases can dramatically alter the conformational ensemble of disordered proteins and thus regulate their function (6,7,15,31), is challenging to characterize with simulations given force field inaccuracies and the long simulation times required to reach convergence for this slow motion. Although NMR generally reports only on ensemble-averaged properties, it provides key insights into the local features of disordered proteins, specifically proline *cis/trans* isomerization, as these slow conversions result in separate NMR signals reporting on the individual proline conformations (14,31).

Using a combination of all-atom explicit solvent metadynamic simulations, and experimental NMR and SAXS, we have accurately characterized both the *cis*-P57 and *trans*-P57 conformational subensembles of the 21-residue ORF6_CTR_ from SARS-CoV-2. This includes an accurate description of many of the features of both *cis-*P57 and *trans-*P57 subensembles, including secondary structure content, *R*_g_, and P57 *cis*/*trans* populations. Applying the *ζ* and *ψ* CVs for P57 in ORF6_CTR_ shows that it is possible to achieve convergence for a process that generally occurs on a timescale of seconds to minutes (12,13) and in a system that is highly computationally expensive to simulate given its heterogeneous FES. While all three force fields used here produced ensembles with *R*_g_, chemical shifts predictions, and secondary structure content that agreed with experimental data, only the a03ws force field generated an ensemble with a *cis-*P57 population that agreed with those calculated from NMR. The a03ws force field appears to agree best with the experimental data in the case of the highly disordered peptide presented above; however, further considerations will be required if simulating other systems with distinct features. Our integrative approach will be particularly relevant for investigating the interactions of disordered protein ensembles with ligands, where proline isomerization can be influenced by binding. In the context of ORF6_CTR_, the proximity of residue P57 to the essential M58 residue (33,34,35) suggests that proline isomerization could play a regulatory role in binding to host proteins. Although the potential influence of proline isomerization has previously been discussed for the isomeric configuration of P57 in the bound-state crystal structure (34), the effects of proline isomerization on binding free energy and potential long-range dynamics remain open questions. We anticipate that the ongoing accumulation of data will further enhance the accuracy of force fields to characterize the *cis-*proline state, and agreement between experimental ensemble-averaged *cis*-proline conformations and MD resolved *cis*-proline conformations will only continue to improve.

## Data and code availability

Code that supports the findings of this study is available from GitHub at https://github.com/hansenlab-ucl/orf6-ctr_cis_trans_conformers and data files are available from Zenodo at https://doi.org/10.5281/zenodo.13748215. NMR chemical shifts have been deposited in the Biological Magnetic Resonance Data Bank (BMRB) (www.bmrb.wisc.edu) under the following accession codes: **52459** for the ORF6_CTR_ *cis*-P57 and *trans*-P57 configurations, and **52460** for the unlabeled NAc-ORF6_CTR_. Additional data related to this paper may be requested from the authors.

## Acknowledgments

The authors acknowledge Carla Molteni, Kresten Lindorff-Larsen, Francesco Pesce, Charlie Buchanan, Carlo Camilloni, and Thomas Löhr for useful discussions. We acknowledge Greg Towers and Morten Andreas Govasli Larsen for providing the GenScript NAc-ORF6_CTR_ peptide. This work used the ARCHER2 UK National Supercomputing Service (https://www.archer2.ac.uk) under the ARCHER2 Pioneer Projects proposal (project name e692). We acknowledge PRACE for awarding us access under the special allocation COVID-19 in the PRACE Call 21, on TGCC Joliot-Curie (project name COVID-1986). We acknowledge Diamond Light Source for time on beamline BL21 under proposal 32676, and we acknowledge Katsuaki Inoue and Nathan Cowieson for their assistance with the sample preparation, beamline experiments, and analysis. A.J.P. was supported by a Biotechnology and Biological Sciences Research Council (BBSRC) UK Research and Innovation (UKRI) funded studentship, BB/T008709/1, with the London Interdisciplinary Biosciences Consortium Doctoral Training Partnership. L.S.N. was supported by the UCL-Birkbeck Medical Research Council (MRC) DTP (MR/N013867/1). The 10.13039/100010269Wellcome Trust is thanked for the award of a PhD studentship to S.M. (109073/Z/15/Z). G.T.H. was supported by Schmidt Science Fellows, Rosaland Franklin Research Fellowship from Newnham College, Cambridge, and a BBSRC Discovery Fellowship (BB/X009955/1). D.F.H was supported by the Engineering and Physical Sciences Research Council (10.13039/501100000266EPSRC) grant (EP/X036782/1). The 10.13039/501100000268BBSRC (BB/R000255/1), 10.13039/100010269Wellcome Trust (ref. 101569/z/13/z), and the 10.13039/501100000266EPSRC are acknowledged for supporting the NMR facility at University College London. Access to ultra-high field NMR spectrometers was supported by the Francis Crick Institute through provision of access to the MRC Biomedical NMR Centre. The Francis Crick Institute receives its core funding from 10.13039/501100000289Cancer Research UK (FC001029), the 10.13039/501100000265UK Medical Research Council (FC001029), and the 10.13039/100010269Wellcome Trust (FC001029). For the purpose of open access, the author has applied a Creative Commons Attribution (CC BY) licence to any Author Accepted Manuscript version arising. This research is supported by the 10.13039/100014013UKRI and 10.13039/501100000266EPSRC.

## Author contributions

A.J.P., G.T.H., and C.D.L. performed and analyzed the metadynamic simulations. A.J.P., V.K.S., and S.M. produced the samples. A.J.P., A.M.F., L.S.N., V.K.S., and D.F.H. performed and analyzed NMR data. A.J.P. analyzed the SAXS data. A.B.T., G.T.H., C.D.L., and D.F.H. designed and supervised the research. All authors discussed the results and wrote the paper.

## Declaration of interests

The authors declare no competing interests.

## Supporting citations

References (76,77,78,79,80,81,82,83,84,85,86,87,88) appear in the supporting material.

## Supporting material

Document S1. Figures S1–S17, Tables S1 and S2, and Equations S1–S11

Document S2. Article plus supporting material

## Footnotes

Supporting material can be found online at https://doi.org/10.1016/j.bpj.2024.09.028.
